# Role of SVEP1 in Stroma-Dependent Hematopoiesis *In vitro*

**DOI:** 10.3389/fcell.2021.760480

**Published:** 2022-01-31

**Authors:** Vinson Tran, Helen C. O’Neill

**Affiliations:** ^1^ Research School of Biology, Australian National University, Canberra, ACT, Australia; ^2^ Clem Jones Centre for Regenerative Medicine, Bond University, Gold Coast, QLD, Australia

**Keywords:** myelopoiesis, stroma, spleen, SVEP1, hematopoietic stem cell

## Abstract

Study of the microenvironment that supports hematopoietic stem cell (HSC) development *in vivo* is very difficult involving small numbers of interacting cells which are usually not well defined. While much is known about HSC niches located within the bone marrow in terms of contributing cell types and signalling molecules, very little is known about equivalent niches within spleen. Extramedullary hematopoiesis in spleen contributes myeloid cells important in the mobilisation of an immune response. As a result, it is important to develop *in vitro* models to identify the cells which constitute HSC niches in spleen and to identify the regulatory molecules supporting myeloid cell development. Studies described here document a model system to study the maintenance and differentiation of HSC by splenic stromal cells *in vitro*. The splenic stromal lines 5G3 and 3B5 differ in hematopoietic support capacity. SVEP1 and IGF2 are molecules of interest specifically expressed by 5G3 stroma. Gene knockdown technology using shRNA plasmids has been used to reduce gene expression in 5G3 and to determine specific effects on myeloid cell development following co-culture with overlaid hematopoietic progenitors *in vitro*. Knockdown of *Svep1* gave specific inhibition of a dendritic cell (DC) population described previously in spleen (L-DC). Knockdown of *Igf2* resulted in loss of production of a minor subset of conventional (c) DC. SVEP1 is now considered a marker of mesenchymal stromal cells with osteogenic differentiative capacity reflective of perivascular stromal cells. The power of this *in vitro* model is evidenced by the fact that it has been used to define SVEP1 as a specific adhesion molecule that regulates the hematopoietic process dependent on stromal niche interaction. The identification of stromal cells and molecules that contribute to the hematopoietic process in spleen, brings us closer to the realm of therapeutically regulating hematopoiesis *in vivo*, and to inhibiting niches which support cancer stem cells.

## Introduction

Hematopoietic stem cells (HSC) are supported by the “stem cell niche,” a tightly regulated environment comprising stromal cells and molecules that control HSC self-renewal, quiescence, differentiation, proliferation and migration (Oh and Kwon, 2010; Raaijmakers, 2010). Stromal adhesion molecules maintain close proximity between stem cells and stromal cells comprising the niche. In adults, the main HSC niche in bone marrow has been described in terms of three interconnected cellular microenvironments, namely the endosteal, perivascular and vascular niches (Kiel and Morrison, 2008; Bianco, 2011; Nagasawa et al., 2011). A combination of cells including osteoblasts, perivascular reticular cells, endothelial cells, macrophages and adipocytes all contribute to the hematopoietic niche in bone marrow and to hematopoietic differentiation (Morrison and Scadden, 2014). The spleen supports extramedullary hematopoiesis and specifically myelopoiesis, and in the steady-state also contains HSC (Wolber et al., 2002; Dor et al., 2006; Tan and O'Neill, 2010; Morita et al., 2011; Hara et al., 2014). In mice, perisinusoidal cells in contact with HSC have been identified in the red pulp region of spleen and described as mesenchymal cells expressing molecules including PDGFR, CXCL12 and KITL which support hematopoiesis (Kiel et al., 2005; Inra et al., 2015; Oda et al., 2018). Stromal cells which function *in vitro* as a splenic niche for HSC have now been described (Tan and O'Neill, 2007; O'Neill et al., 2011).

Long-term cultures (LTC) comprising splenic stroma were developed in this lab in order to investigate potential hematopoietic niche elements in spleen. These cultures reflect an *in vitro* microenvironment which supports restricted myelopoiesis with continuous production of progenitors, myeloid/dendritic cells, and a novel dendritic-like cell type, referred to as “long-term culture-derived dendritic cells” or “L-DC” (Wilson et al., 2000; O'Neill et al., 2004). The long-term production of L-DC, but not other cell types, was found to be dependent on stromal cell contact (Wilson et al., 2000; Periasamy et al., 2013). The possibility that HSC in spleen arise from endogenous progenitors laid down during embryogenesis, as opposed to bone marrow-derived progenitors entering spleen through blood, has also been considered (O'Neill et al., 2004; O'Neill et al., 2011).

In order to investigate stromal elements supporting hematopoiesis, stroma was isolated and cloned from the STX3 spleen LTC that had ceased production of cells due to loss of progenitors over time and passage (Despars et al., 2004). The overlay of lineage negative (Lin^-^) bone marrow cells on to STX3 stroma in co-cultures led to myelopoiesis and production of cells very similar to LTC (Periasamy et al., 2009). These stromal co-cultures produce progenitors, myeloid cells/precursors and dendritic-like cells including the novel L-DC subset (Periasamy et al., 2009; Periasamy et al., 2013; Periasamy and O'Neill, 2013; Petvises and O'Neill, 2014). Analysis of over a hundred distinct clonal lines derived from STX3 identified 5G3 as a rapidly growing clone which was also a supporter of hematopoiesis. 3B5 was selected as a non-supporter (Despars and O'Neill, 2006a). Further studies questioned which hematopoietic stem/progenitor cell (HSPC) subsets gave rise to L-DC. This study employed known progenitor subsets from bone marrow including long-term HSC (LT-HSC) with self-renewal capacity (Kiel et al., 2005), as well as multipotential progenitors (MPP) with more limited self-renewal capacity and high differentiative potential (Christensen and Weissman, 2001). Both gave rise to L-DC when overlaid on 5G3 in co-cultures such that both subsets appear to contain distinct L-DC progenitors which may be developmentally linked (Petvises and O’Neill, 2014a). Other less well defined progenitors tested included myeloid/dendritic progenitors [MDP] and the common dendritic progenitor (CDP) downstream of the common myeloid progenitor (CMP) (Onai et al., 2007; Liu et al., 2009), but these produced dendritic cells (DC) resembling conventional (c) DC or myeloid DC (Petvises and O’Neill, 2014a). This evidence clearly defined the lineage origin of L-DC as distinct from other dendritic and myeloid cells, and identified a self-renewing HSC as the L-DC progenitor. Furthermore, L-DC development was shown to occur independently of macrophage-colony stimulating factor (M-CSF/CSF1), FMS-like tyrosine kinase-3 ligand (Flt3L) or granulocyte macrophage-colony stimulating factor (GM-CSF) (Petvises and O’Neill, 2014a) which are known regulators of myelopoiesis (Onai et al., 2007; Xu et al., 2007).

A number of splenic stromal cell lines isolated from STX3, including 5G3 and 3B5, have been shown to have similar cell surface marker profile reflecting mesenchymal stem cells (MSC) since they express known markers including CD29, VCAM1, LY6A and Thy1.2 (Lim et al., 2018; O’Neill et al., 2019). They also closely resemble a perivascular subset of MSC in bone marrow known as CXCL12- abundant reticular (CAR) cells due to their marker expression of CD51 and CD140A (Omatsu et al., 2010; Pinho et al., 2013). Further evidence that 5G3 and 3B5 reflect a perisinusoidal/perivascular reticular cell type is ability to undergo osteogenesis when cultured under mineralisation conditions (Askarinam et al., 2013; O’Neill et al., 2019). The functional role of 5G3 as a hematopoietic niche is supported by data from transcriptome analysis showing that 5G3 expresses genes which regulate hematopoiesis, including *Cxcl12*, *Vcam1* and *Spp1* (Periasamy et al., 2018). Inhibition of the CXCL12 and VCAM1 signalling in 5G3 co-cultures was found to give a general reduction in cell production (Lim et al., 2018; Periasamy et al., 2018) consistent with the literature which shows that stromal cells in hematopoietic niches produce CXCL12 that binds to CXCR4 (C-X-C chemokine receptor type 4) and CXCR7 (C-X-C chemokine receptor type 7) on HSC, so directing cells to the stromal cell surface and signalling their differentiation (Sugiyama et al., 2006; Sánchez-Martín et al., 2013). Once HSC come into close proximity with the stromal niche, adhesion molecules like VCAM1 interact with VLA-4 expressed by HSC, so allowing HSC to adhere to stroma and to receive signals which support their maintenance and differentiation (Ulyanova et al., 2005; Martinez-Agosto et al., 2007). The blocking of SPP1 binding to CD44 was shown to specifically decrease L-DC production and to restrict the HSC pool by maintaining HSC in a quiescent state (Periasamy et al., 2018), consistent with the previously published role of SPP1 in bone marrow niches (Nilsson et al., 2005; Stier et al., 2005). Inhibition of interactions like these between stroma and HSC may lead to exhaustion of the HSC pool, so affecting the later production of differentiated cells.

Despite the similar lineage origin of 5G3 and 3B5, only 5G3 is a supporter of *in vitro* hematopoiesis, and specifically of L-DC development. Transcriptome analysis was used to identify genes specifically expressed by stromal lines like 5G3 over 3B5. This study identified a number of genes specifically expressed by 5G3 which are candidates for hematopoietic support. Genes investigated here include *Svep1* (sushi, von Willebrand factor type A, EGF and pentraxin containing 1) and *Igf2* (insulin-like growth factor 2) (Periasamy et al., 2018). Of particular interest is SVEP1, a selectin-like molecule identified as specifically and highly expressed by 5G3, but for which no specific antibodies exist. Integrin α_9_β_1_ is a known ligand for SVEP1 (Sato-Nishiuchi et al., 2012), and antibody to integrin α_9_β_1_ inhibited production of cells in 5G3 stromal co-cultures (Periasamy et al., 2018). However, VCAM1 is also a receptor for integrin α_9_β_1_ (Ross et al., 2006; Saldanha-Gama et al., 2010). In order to confirm a role for SVEP1 in *in vitro* hematopoiesis and L-DC development, it was therefore necessary to knockdown *Svep1* expression in 5G3 cells.

This work is further justified by recent reports which associate SVEP1 with early hematopoiesis. Evidence favouring a role for SVEP1 in hematopoiesis comes from transcriptomic analysis of the embryonic aorta, which shows conservation across species of molecules which regulate HSC, one of which is SVEP1 (Yvernogeau et al., 2020). Furthermore, a subset of mesenchymal stem/stromal cells in human bone marrow has been identified through expression of low affinity nerve growth factor receptor (CD271) (Kuçi et al., 2010; Kuçi et al., 2019). This subset of stroma is distinct through expression of genes supporting hematopoiesis including *Cxcl12*, *Flt3l*, *Il-3*, *Tpo* and *KitL*, and also through specific expression of several adhesion molecules including *Svep1* (Kuçi et al., 2019).

Here, gene knockdown in splenic stromal lines has been used to specifically test the role of genes in *in vitro* hematopoiesis in stromal co-cultures. Several strongly expressed genes including *Svep1*, *Igf2* and *Csf1*, have been knockdown in 5G3 stroma using short hairpin RNA (shRNA) plasmids. Co-cultures were then established using sorted subsets of HSPC as overlays above knockdown stroma.

## Materials and Methods

### Tissue Culture

Cells were cultured at 37°C in 5% CO_2_ in air in Dulbecco’s modified Eagle’s medium (DMEM) supplemented with 10% fetal calf serum, 5 × 10^−4^M 2-mercaptoethanol, 10mM HEPES, 100U/ml penicillin, 100ug/ml streptomycin, 4 g/l glucose, 6 mg/l folic acid, 36 mg/l L-asparagine, 116 mg/l L-asparagine hydrochloric acid (sDMEM).

The 5G3 and 3B5 stromal lines were cloned from STX3 splenic stroma derived from a long-term culture of spleen from B10. A (2R) mice (H-2K^k^) (Despars et al., 2004; Despars and O'Neill, 2006a). The original stromal cell lines, as well as transfected stromal lines, were grown from frozen stocks and passaged up to five times by transfer of cells to new flasks following trypsinization using 0.25% trypsin-EDTA treatment to dissociate cells. Stromal cells in culture were maintained by passage every 4 days by scraping and transferring cells to a new flask (Periasamy et al., 2009).

The BCL1 B cell line and P815 mastocytoma cell line were cultured in sDMEM and passaged every 3–4 days.

### Animals

Specific pathogen-free C57BL/6J (*H-2K* ^*b*^) mice at 6 weeks of age were obtained from the John Curtin School of Medical Research (JCSMR: Canberra, ACT, Australia). Mice were housed and handled according to protocols approved by the Animal Experimentation Ethics Committee at the Australian National University (ANU: Canberra, ACT, Australia).

### Preparation of Murine Cells

Mice were euthanized, followed by sterile dissection of tissues. Cell suspensions were dissociated by forcing tissue through a fine wire sieve. Lysis of red blood cells involved hypertonic treatment as described previously (Petvises and O'Neill, 2014). For separation of stromal cells from leukocytes in spleen, thymus and lymph node, the non-suspendable stromal fraction was treated with collagenase IV (1 mg/ml) and DNase (40 μg/ml) in DMEM, with incubation at 37°C for 20 min and slow rotation. This was followed by two further treatments with DMEM containing collagenase D (1 mg/ml) and DNase (40 μg/ml) with slow rotation for 20 min. Collagenase was then inactivated by addition of EDTA (500 mM). Stromal cells were then washed by centrifugation, passed through a 100 μm cell strainer, and resuspended into medium.

Bone marrow was flushed from the bone cavity with sDMEM. Bone marrow was depleted of Lin^+^ cells using a lineage depletion antibody kit supplemented with antibody to CD11c (Miltenyi Biotec, Bergisch Gladbach, GL, Germany) and MACS^®^ magnetic bead technology (Miltenyi Biotec) as described previously (Periasamy et al., 2009; Periasamy et al., 2013). Over multiple separations, efficiency of depletion was shown to be ∼95%.

In preparation for osteogenic differentiation, bone marrow cells were cultured at 10^7^ cells/mL in sDMEM. After 24 h, medium containing non-adherent cells was removed and replaced. Adherent MSC were maintained in cultures by medium replacement every 3 days. After 14–18 days, cells were dissociated using 0.25% trypsin-EDTA treatment for 2 min at 37°C. Cells were then plated at a concentration of 10^5^ cells/mL in preparation for culture under conditions that stimulate osteogenesis.

### Osteogenic Differentiation

Cultures of bone marrow-derived MSC, or of 5G3 and 3B5 stroma, were maintained for up to 4 weeks in sDMEM, containing 10^−8^ M dexamethasone, 100 μM ascorbic acid 2-phosphate (AA2P) and 10 mM β-glycerophosphate to induce mineralisation or osteogenesis (O’Neill et al., 2019). Medium was replaced every 4 days. 5G3 and 3B5 cultures were maintained at a concentration of 10^5^ cells/mL by passaging cells every 4 days using 0.25% trypsin-EDTA treatment to dissociate cells. Parallel cultures were maintained in sDMEM medium as undifferentiated control cells. After 8, 16 and 24 days of culture under mineralization conditions, RNA was prepared and qRT-PCR performed.

### Establishment of Co-Cultures

Stromal cell lines were grown as a monolayer to 80–90% confluency. Lin^-^ bone marrow cells were added at 1-5 x 10^4^ cells/ml as an overlay. Progenitor cells sorted from bone marrow were plated at 10^3^ cells/5ml/flask. Co-cultures were held at 37°C, 5% CO_2_ in air and 97% humidity. Medium changes were performed every 3–4 days by removal of half volume and replacement with sDMEM. At 7-day intervals, non-adherent cells were collected through removal and replacement of supernatant. Cell yield was determined and cell subsets identified through analysis of surface marker expression by antibody staining and flow cytometry.

### Flow Cytometry

The procedure used to stain cells with multiple fluorochrome-conjugated antibodies has been described in detail previously (Petvises and O'Neill, 2014; Petvises and O’Neill, 2014a). “Fc block” specific for FcγII/IIIR (eBioscience, Parkville, VIC, Australia) was absorbed to cells ahead of antibody to block non-specific Fc receptor binding. Antibodies were purchased from Biolegend (San Diego, CA, United States). Those used to stain murine myeloid cells were specific for CD11c (N418), CD11b (M1/70), MHC-II(AF6-120.1) and F4/80 (A3-1). Antibodies used to stain bone marrow progenitors for sorting were specific for Sca-1 (D7, PB), cKit (2B8), Flt3 (A2F10), CD150 (TC15-12F12.2) and CD115 (AFS98). Dead cell discrimination involved addition of 1 μg/ml of propidium iodide (PI) to cells prior to flow cytometric analysis. To stain lineage (Lin)^+^ cells for gating during flow cytometry, a lineage depletion antibody kit supplemented with antibody to CD11c was employed (Miltenyi Biotec). Flow cytometry was performed on an LSRII FACS machine (Becton Dickinson: Franklin Lakes, NJ, United States). Voltage, parameter and event counts were programmed using BD FACSDIVA software (Becton Dickinson). Single colour controls were used to set compensation. FlowJo® software (Ashland, OR, United States) was used to analyse data. Live cells were gated by the absence of PI staining (PI^-^), and then gated on the basis of forward scatter (FSC) and side scatter (SSC). Fluorescence-minus-one controls (FMOCs) were used to set gates to distinguish specific antibody binding.

### Microscopy

Photographs of stromal cells were taken using an EVOS® FL digital fluorescence microscope (Electron Microscope Sciences: Hatfield, PA, United States), equipped with a Sony® ICX445 CCD camera (Sony: Minato, TKY, JP). Fluorescent micrographs were taken using a Leica TCS SP5 Confocal microscope (Leica Microsystems: Weitziar, HE, GER) at 40X magnification.

### Use of SmartFlare ™ Probes for RNA Detection

SmartFlare™ probes (Merck Millipore: Billerica, MA, United States) comprise a gold nanoparticle bound to multiple capture strands. A reporter strand that carries a fluorescent “flare” is hybridized to the capture strand. Upon endocytosis by cells of interest, target mRNA binds to the capture strand, so displacing the reporter strand, which can be detected by its ability to fluoresce once removed from the quenching influence of the gold nanoparticle (Seferos et al., 2007). Probes were obtained from Merck Millipore and included *Actb* Cy5 (SF-781) and *Svep1* Custom Cy5 (SFC-565), a Scrambled Target Control Cy5 (SF-102) and an Uptake Control Cy5 (SF-137) to act as negative and uptake controls, respectively.

Cells of interest were plated at 80% confluency (3 × 10^5^ cells/200 μL) in sDMEM medium. The SmartFlare^TM^ reagent was diluted in sterile PBS to a concentration of 500 pM, and 4 μL added to each well containing cells. Cultures were incubated for up to 24 h at 37°C, 5% CO_2_ and 95% humidity. Fluorescent cells were detected through flow cytometric analysis or Confocal microscopy.

### Quantitative Realtime-Polymerase Chain Reaction

RNA was extracted from stromal cells using the Qiagen RNeasy minikit (SABiosciences, Valencia, CA, United States) and reverse transcribed into cDNA using the RT^2^ First Strand Synthesis kit (SABiosciences) as described previously (Periasamy et al., 2018). Equal amounts of cDNA and primers (10uM) were used. Primers were purchased from SABiosciences: *Svep1* (PPM05259A), *Actb* (PPM02945A), *Csf1* (PPM03116C), *Igf2* (PPM03655A) and *Ms4a4d* (PPM24747A). cDNA and primer mix were added to RT^2^ SYBR Green Mastermix and RNase-free water in a ratio of 1:6.25:5.25. Samples were then run in a LightCycler 480 (Roche, Penzberg, BAV, Germany). A single run involved: 1 cycle of 10 min at 95°C to activate polymerase, 45 cycles of 15 s at 95°C for extension, and 1 min at 60°C for detection of fluorescence.

Data analysis involved LightCycler 480 software v. 1.2.9.11 (Roche). To obtain a cross point value (C_p_), also referred to as the cycle threshold (C_t_), the Absolute Quantification (second derivative max) method was used. C_p_ is the point where maximal increase in fluorescence emitted by a single PCR reaction within the log-linear phase occurs. C_t_ values for genes of interest (GOI) along with housekeeping genes (HKG) were imported into Excel (Microsoft: Redmond, WA, United States) for further analysis. Change in ΔC_t_ = C_t_ (GOI)—C_t_ (HKG) was calculated, and the average ΔC_t_ taken from quadruplicate experiments. To calculate the fold change between two samples, the calculation 2^−ΔCt^ (Sample 1)/2^−ΔCt^ (Sample 2), was used. The resulting value corresponds to the relative difference in mRNA quantity between two samples for a GOI. The presence of an amplified product was confirmed by gel electrophoresis.

### Transfection of Cells With shRNA

5G3 was transfected with shRNA to establish knockdown lines. The pLKO.1-puro plasmid vector (Sigma-Aldrich: St Louis, MO, United States) contains both ampicillin resistance *AmpR* and puromycin resistance *PurR* genes for selection of bacterial and mammalian cell transfectants, respectively. The sensitivity of 5G3 to puromycin was assessed initially in order to identify the minimum effective concentration of drug and treatment times for stromal cells.

All shRNA plasmids were purchased from Sigma-Aldrich. Each of the shRNA was supplied as glycerol stocks of transformed bacteria. These included: *Svep1* [TRCN0000351057 (shRNA1); TRCN0000340274 (shRNA2); TRCN0000340211 (shRNA3); TRCN0000340213 (shRNA4); TRCN0000340212 (shRNA5)], *Csf1* [TRCN0000065908 (shRNA1); TRCN0000065909 (shRNA2); TRCN0000065910 (shRNA3); TRCN0000065911 (shRNA4); TRCN0000065912 (shRNA5)], and *Igf2* [TRCN0000071147 (shRNA1); TRCN0000071149 (shRNA2); TRCN0000071150 (shRNA3)]. The control vector was supplied as a plasmid, and was firstly transformed into *E. coli* JM109 and plated out on agar containing Carbenicillin (CB; 0.1 mg/ml) to select transformants. Bacterial glycerol stocks of shRNA were streaked on CB agar plates. A single colony from each of the control and shRNA CB agar plates was inoculated into Luria broth (LB) containing 0.1 mg/ml of CB. A plasmid miniprep kit (Pureyield^™^ plasmid miniprep system; Promega: Madison, WI, United States) was used to prepare plasmid DNA for frozen storage according to the manufacturer’s instructions.

On the day prior to transfection, 8 × 10^4^ cells were plated in 500 μL sDMEM with overnight incubation. Transfection mix for each shRNA plasmid, comprised 400 ng of DNA/60 μL sDMEM. Attractene Transfection Reagent (Qiagen: Venio, LI, NA) (1.5 μL) was added to each transfection mix and incubated at 20°C for 20 min. Medium on cells was replaced, and the cells allowed to recover through incubation for 24 h. Transfectants were then selected for 72 h by replacing medium with 1 ml of sDMEM containing 1 μg/ml puromycin. Cells were then grown to 100% confluency with sDMEM changes every 24 h.

### Statistical Analysis

When replicates could be prepared, data are presented as mean ± S.E. for sample size n. The Wilcoxon Rank Sum test was used to assess significance (*p* ≤ 0.05).

In co-culture experiments where only low numbers of progenitors are seeded and cell production is low, cumulative cell production was measured at several time points in preference to replication of cells produced at one time point. A significant effect is indicated by increasing cell production across 4 time points. The null hypothesis is that cell production is random over time, and the alternative hypothesis is that increasing cell production occurs with increasing time. The probability of an ordered expanding sequence for cell production over 4 time points is 1/24 or 0.0417 which reflects significant cell production (*p* ≤ 0.05).

## Results

### Spleen Stromal Lines Support *In vitro* Hematopoiesis

**FIGURE 1:**
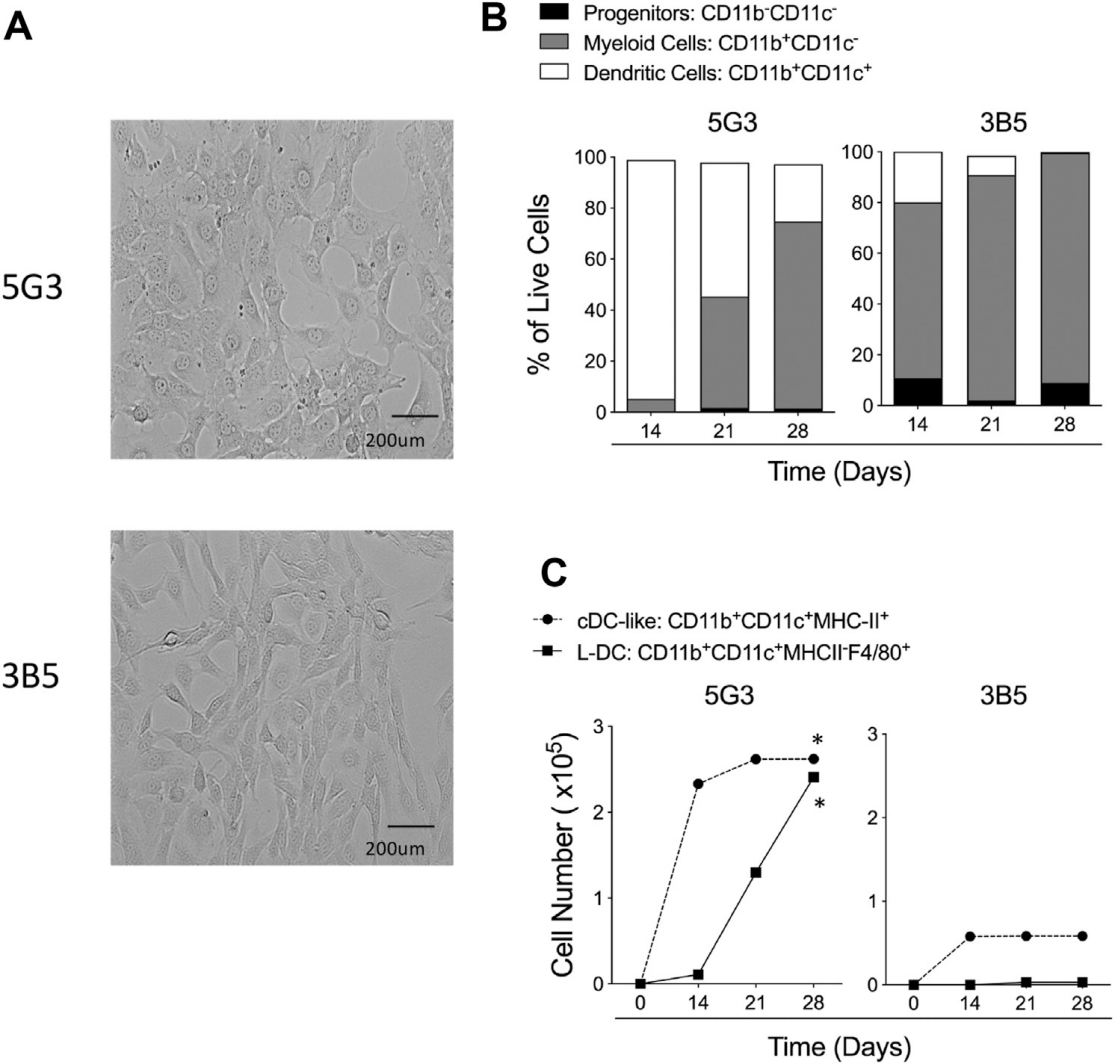
Hematopoietic support capacity of splenic stroma. 5G3 and 3B5 are distinct stromal cell lines cultured to confluence over 28 days **(A)**. Lin^-^ bone marrow was co-cultured above stroma for 28 days and non-adherent cells collected at medium change weekly for flow cytometric analysis of cell types present. Antibodies used were specific for CD11c, CD11b, F4/80 and MHC-II in order to gate broad subsets of CD11b^-^CD11c^-^ progenitor cells, CD11b^+^CD11c^-^ myeloid cells and CD11b^+^CD11c^+^ DC. Proportion of each of these subsets amongst live (PI^-^) non-adherent cells over time in shown in **(B)**. DC were further gated as MHC-II^+^ cDC-like cells, and MHC-II^-^F4/80^+^ L-DC. Data are presented as cumulative cell production over time. Cultures showing significant (*p* = 0.0417) increasing subset production over 4 time points are shown by *(C).

The 5G3 and 3B5 splenic stromal lines reflect morphologically distinct clonal lines of similar lineage origin cloned from stroma isolated from a single spleen LTC (Despars and O'Neill, 2006b; Periasamy et al., 2018) (Figure 1A). The two stromal lines differ in terms of hematopoietic support capacity after establishment of co-cultures with overlaid Lin^-^ bone marrow cells (Figures 1B,C). Non-adherent cells were collected on Days 14, 21 and 28, and antibody staining and flow cytometric analysis used to distinguish subsets amongst cells produced over time. Here, progenitors were identified as CD11b^-^CD11c^-^, myeloid cells/precursors as CD11b^+^CD11c^-^, cDC-like cells as CD11b^+^CD11c^+^MHC-II^+^ and L-DC as CD11b^+^CD11c^+^MHC-II^-^F4/80^+^. The clear identification of the novel L-DC subset on the basis of F4/80 staining was recently introduced into subset analyses (Petvises and O'Neill, 2014).

All data show that 5G3 is a stronger supporter of hematopoiesis than 3B5, and also produces L-DC, while 3B5 does not. 3B5 co-cultures produced more myeloid cells than DC (Figure 1B), but with lower cell production compared with 5G3 (Figure 1C). Only 5G3 showed significant production of L-DC and numbers increased over 28 days (Figure 1C). Both 5G3 and 3B5 co-cultures produced cDC-like cells initially, but production was transient and decreased over time (Figure 1C).

### *Svep1* Gene Knockdown in 5G3

The gene knockdown procedure was optimised to determine the functional importance of SVEP1 in the hematopoietic support function of 5G3 stroma. Other genes investigated included *Igf2* also expressed by just 5G3, as well as *Csf1* which is strongly expressed by both cell lines. Transfection with shRNA plasmids was chosen over siRNA since shRNA allows generation of stable knockdown cell lines and is more suited to continuously growing lines. Furthermore, the concentration of shRNA remains stable as cells divide so maintaining a constant gene knockdown effect (Taxman et al., 2010).

**FIGURE 2:**
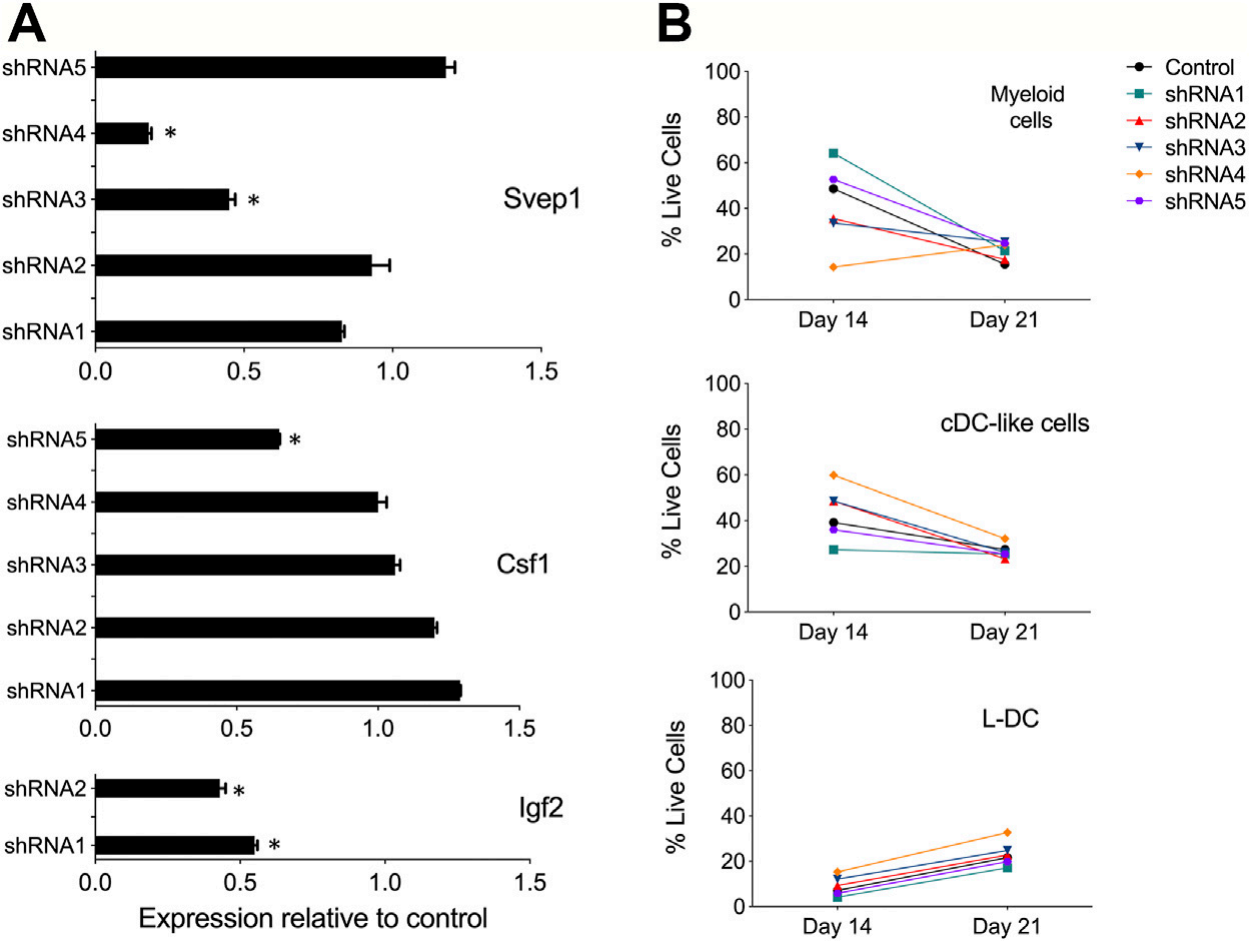
Evidence for gene knockdown in 5G3 stroma. **(A)** Knockdown plasmids containing up to 5 distinct shRNA specific for *Svep1*, *Csf1* or *Igf2*, or an empty plasmid vector as control, were transfected into 5G3 followed by 3 days of puromycin selection (1 mg/ml). Cells were collected and RNA prepared for qRT-PCR to detect changes in gene expression. Data represent gene expression measured by qRT-PCR for each of the shRNA transfected lines relative to the control. Data represent mean ± S.E. from 4 independent PCR reactions. * identifies gene expression significantly less than control (*p* ≤ 0.05). **(B)** Hematopoietic support capacity of confluent cultures of each of the *Svep1* knockdown lines, and the control line, was tested by capacity for cell production from overlaid Lin^-^ bone marrow cells. Non-adherent cells were collected on Days 14 and 21 of co-culture. Cells were counted and stained with fluorochrome-conjugated antibodies specific for CD11b, CD11c, MHC-II and F4/80. “Fluorescence minus one” controls were used to set gates to identify specific antibody binding. All co-cultures produced equal numbers of cells. Subsets were identified as CD11b^+^CD11c^-^ myeloid cells, CD11b^+^CD11c^+^MHC-II^+^ cDC-like cells, and CD11b^+^CD11c^+^MHC-II^-^ L-DC. Data are presented as % of each cell type produced in co-cultures.

Multiple *Svep1* shRNA plasmids were tested for each gene. The shRNA plasmids and a control (empty vector) plasmid were transfected into separate cultures of 5G3. qRT-PCR was performed on all transfectants and compared with the control (empty vector) transfectants after 7 days to verify gene knockdown. For *Svep1*, cell lines transfected with shRNA3 and shRNA4 showed significant reduction in *Svep1* expression, with values of gene expression relative to control of 0.45 and 0.18, respectively (Figure 2A). For *Csf1* transfections, the greatest knockdown effect was seen with shRNA5 (0.65), and for *Igf2* transfection, both shRNA1 and shRNA2 were very effective with expression relative to control of 0.45 and 0.55, respectively (Figure 2A).

Initially, selected *Svep1* shRNA transfected lines and the control transfected (empty vector) line were cultured as a monolayer and then overlaid with Lin^-^ bone marrow to observe any effect of knockdown on the ability of 5G3 to support hematopoiesis. This was assessed in terms of production of myeloid and dendritic subsets through flow cytometry. Myeloid cells and precursors were identified after 14 and 28 days as a CD11b^+^CD11c^-^ subset, cDC-like cells as CD11b^+^CD11c^+^MHC-II^+^ and L-DC as CD11b^+^CD11c^+^MHC-II^-^. Apart from some partial changes in number of cells produced in co-cultures using shRNA4 transfectants, cell production was relatively constant across all cultures (Figure 2B). While the shRNA protocol was effective in knocking down of *Svep1* expression in 5G3, this change did not dramatically alter the hematopoietic support function of 5G3 in Lin^-^ bone marrow co-cultures. Similar results were also obtained for *Csf1* and *Igf2* knock down stromal lines (data not shown).

### Gene Knockdown in Stromal Co-Cultures of Hematopoietic Progenitors

**FIGURE 3:**
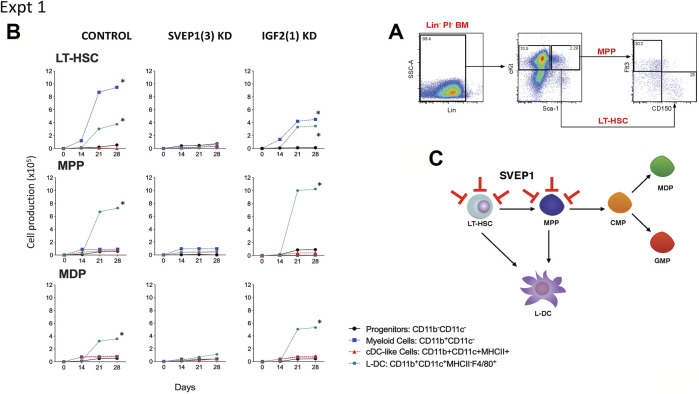
Effect of *Svep1* knockdown on 5G3 hematopoietic support capacity. **(A)** The sorting strategy to isolate LT-HSC and MPP is shown. Lin^-^ bone marrow cells were prepared by antibody depletion using MACS^®^ column technology and anti-biotin microbeads (Miltenyi Biotec). For sorting progenitor subsets, cells were stained with lineage antibodies as well as fluorochrome-conjugated antibodies specific for progenitors: Sca-1, cKit, Flt3, and CD150. Cells were gated initially as Lin^-^ PI^-^ (live) cells. “Fluorescence minus one” controls were used to set gates to identify specific antibody binding. LT-HSC were gated as cKit^+^Sca-1^+^Flt3^−^CD150^+^ cells, and MPP as cKit^+^Sca-1^+^Flt3^+^CD150^-^ cells. **(B)** Co-cultures were established by overlay of equal numbers (1 × 10^3^ cells/flask) of sorted LT-HSC and MPP above control 5G3 stroma, *Svep1* knockdown stroma (SVEP1 (3) KD), and *Igf2* knockdown stroma (IGF2 (1) KD). Non-adherent cells were collected weekly and stained with fluorochrome-conjugated antibodies specific for CD11c, CD11b, MHC-II and F4/80 to identify subsets of CD11b^-^CD11c^-^ progenitor cells, CD11b^+^CD11c^-^ myeloid cells, CD11b^+^CD11c^+^MHC-II^+^ cDC-like cells and CD11b^+^CD11c^+^MHC-II^-^ L-DC. Data are presented as cumulative cell production over time. Cultures showing significant (*p* = 0.0417) increasing subset production over 4 time points are shown by *. **(C)** A model for the role of *Svep1* in hematopoiesis is shown. Red bars show blocking effects of *Svep1* knockdown on LT-HSC and MPP.

**FIGURE 4:**
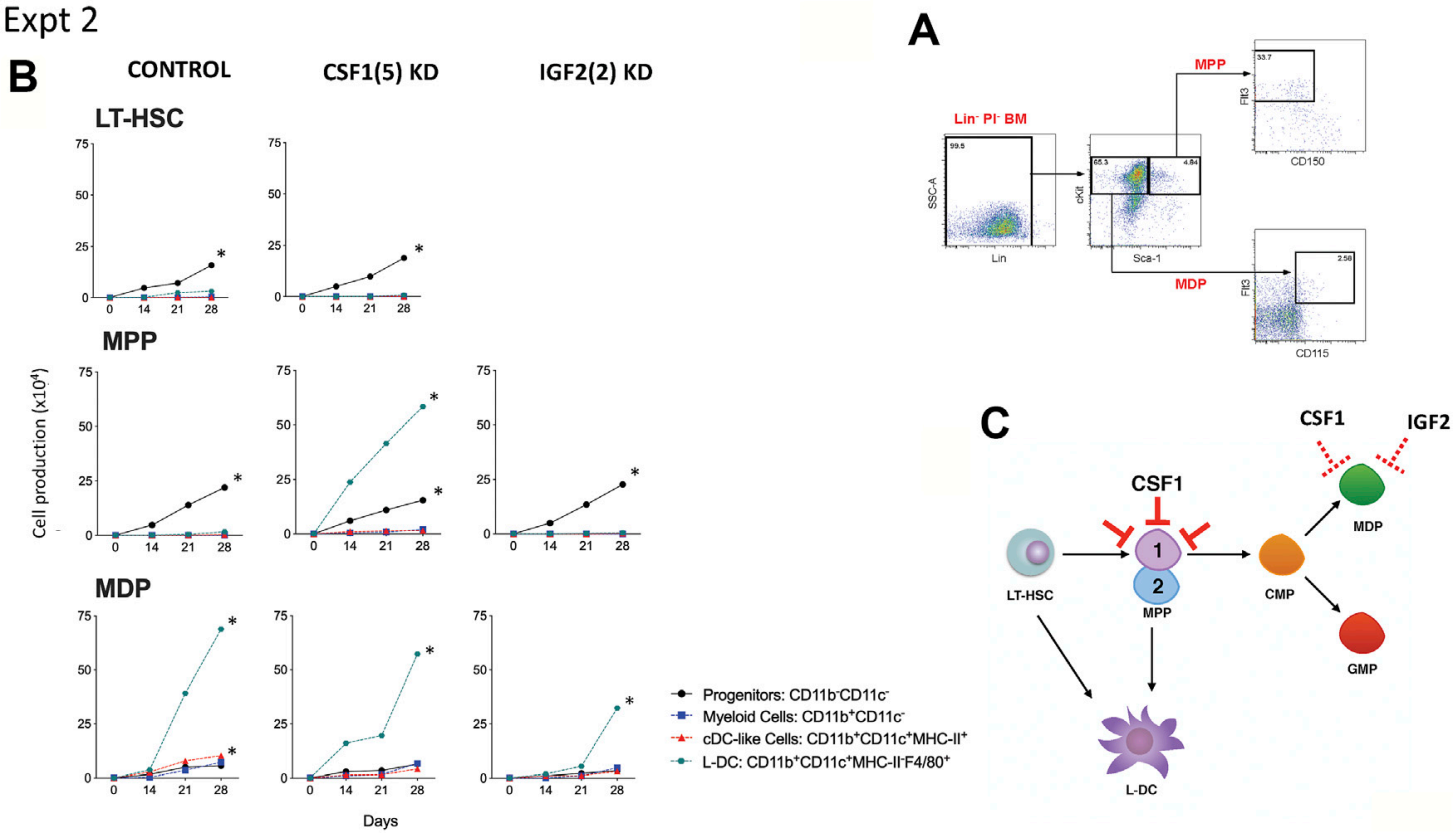
Effect of *Csf1* knockdown on 5G3 hematopoietic support capacity. **(A)** The sorting strategy to isolate MPP and MDP is shown. Lin^-^ bone marrow cells were prepared by antibody depletion using MACS^®^ column technology and anti-biotin microbeads (Miltenyi Biotec). For sorting progenitor subsets, cells were stained with lineage antibodies as well as fluorochrome-conjugated antibodies specific for progenitors: Sca-1, cKit, Flt3, CD150 and CD115. Cells were gated initially as Lin^-^ PI^-^ (live) cells. “Fluorescence minus one” controls were used to set gates to identify specific antibody binding. MPP were gated as cKit^+^Sca-1^+^Flt3^+^CD150^-^ cells, and MDP were further gated as cKit^+^Sca-1^-^Flt3^+^CD115^-^ cells. **(B)** Co-cultures were established by overlay of equal numbers (1 × 10^3^ cells/flask) of sorted MDP and MPP above control 5G3 stroma, *Csf1* knockdown stroma (CSF1(5) KD), and *Igf2* knockdown stroma (IGF2 (2) KD). Non-adherent cells were collected weekly and stained with fluorochrome-conjugated antibodies specific for CD11c, CD11b, MHC-II and F4/80 to identify subsets of CD11b^-^CD11c^-^ progenitor cells, CD11b^+^CD11c^-^ myeloid cells, CD11b^+^CD11c^+^MHC-II^+^ cDC-like cells and CD11b^+^CD11c^+^MHC-II^-^ L-DC. Data are presented as cumulative cell production over time. Cultures showing significant (*p* = 0.0417) increasing subset production over 4 time points are shown by *. **(C)** A model for the role of *Csf1* in hematopoiesis is shown. Red bars show blocking effects of *Csf1* knockdown on one of two subsets of MPP.

One hypothesis is that the co-culture of a heterogeneous population of Lin^-^ bone marrow cells may mask any specific effect of gene knockdown on particular hematopoietic progenitors. Co-cultures were therefore established with knockdown stromal lines overlaid with highly purified LT-HSC sorted as Lin^-^Sca1^+^ckit^+^Flt3^−^CD150^+^ cells (Kiel et al., 2005), the broad MPP subset of Lin^-^Sca1^+^ckit^+^Flt3^+^CD150^-^ cells (Christensen and Weissman, 2001), and the subset of Lin^-^Sca1^+^ckit^-^Flt3^+^CD115^+^ cells which includes MDP (Petvises and O'Neill, 2014). Cell sorting procedures are shown in Figures 3A, 4A. The number of cells available for establishment of co-cultures was very low so that optimisation of conditions was needed to obtain a fully controlled experiment. Preliminary investigations showed that single larger (25 ml) cultures were more supportive of cell production than replicate smaller cultures, so that replication of distinct controlled experiments was chosen over replication within experiments. Single co-cultures were established for each transfected line, using equal numbers of cells and were maintained equivalently over 28 days through weekly medium change and cell collection. The long-term nature of experiments also precluded comparison of outcomes across experiments since sorted cells vary with preparation, and co-culture differences are amplified over such a long culture period.

Two similar experiments are shown here. In the first, LT-HSC, MPP and MDP were sorted from bone marrow and overlaid on 5G3 transfected with the control (empty) vector, the *Svep1* shRNA3 knockdown stroma and the *Igf2* shRNA1 knockdown stroma. Control co-cultures seeded with LT-HSC showed significant increasing production of CD11b^+^CD11c^-^ myeloid cells, most likely reflecting myeloid progenitors and precursors, along with the CD11b^+^CD11c^+^MHCII^-^F4/80^+^ L-DC subset (Figure 3B). Significant cell production was determined by the production of increasing numbers of a cell subset across 4 time points in the assay (*p* = 0.0417). Co-cultures established with the MPP and MDP populations showed significant production of only the novel L-DC subset. The *Svep1* knockdown line showed no significant cell production of any cell type, implicating a role for the SVEP1 protein in myelopoiesis involving the production of both L-DC and myeloid progenitors/precursors. The *Igf2* knockdown line gave similar results as the control, indicating no effect of IGF2 on cell production. Knockdown of *Svep1* can block the development of myeloid cells from progenitors within the sorted LT-HSC and the MPP subsets (Figure 3C), each of which has been shown previously to contain a direct progenitor of the L-DC subset (Petvises and O'Neill, 2014).

A second experiment compared the effects of *Csf1* and *Igf2* knockdown. LT-HSC gave rise to significant production of progenitor cells which was not blocked by knockdown of *Csf1*. A similar finding was reported previously (Petvises and O’Neill, 2014a). MPP co-cultured over control stroma also gave significant production of myeloid progenitors in this experiment, which was not lost through either *Csf1* or *Igf2* knockdown (Figure 4B). *Csf1* knockdown stroma however supported significant production of L-DC. The best explanation for this result is that L-DC progenitors are a subset of MPP and are not the cells dependent on CSF1 (Figure 4C), so that when this gene is knocked down, there is a rebound effect with increased significant production of L-DC (Figure 4B). In contrast, MDP co-cultures gave significant production of only the L-DC subset which was not inhibited by knockdown of either *Csf1* or *Igf2* in 5G3 stroma (Figure 4B). The production of L-DC, suggests the presence of an L-DC progenitor within the sorted MDP population (Lin^-^Sca1^+^ckit^-^Flt3^+^CD115). In Experiment 2, significant but low production of cDC-like cells was evident in control MDP co-cultures which was not lost from the *Csf1* and *Igf2* knockdown co-cultures. This suggests a role for CSF1 and IGF2 in the production of cDC-like cells in 5G3 co-cultures.

### Use of SmartFlare™ Technology to Detect *Svep1*-Expressing Cells in Murine Tissues

**FIGURE 5:**
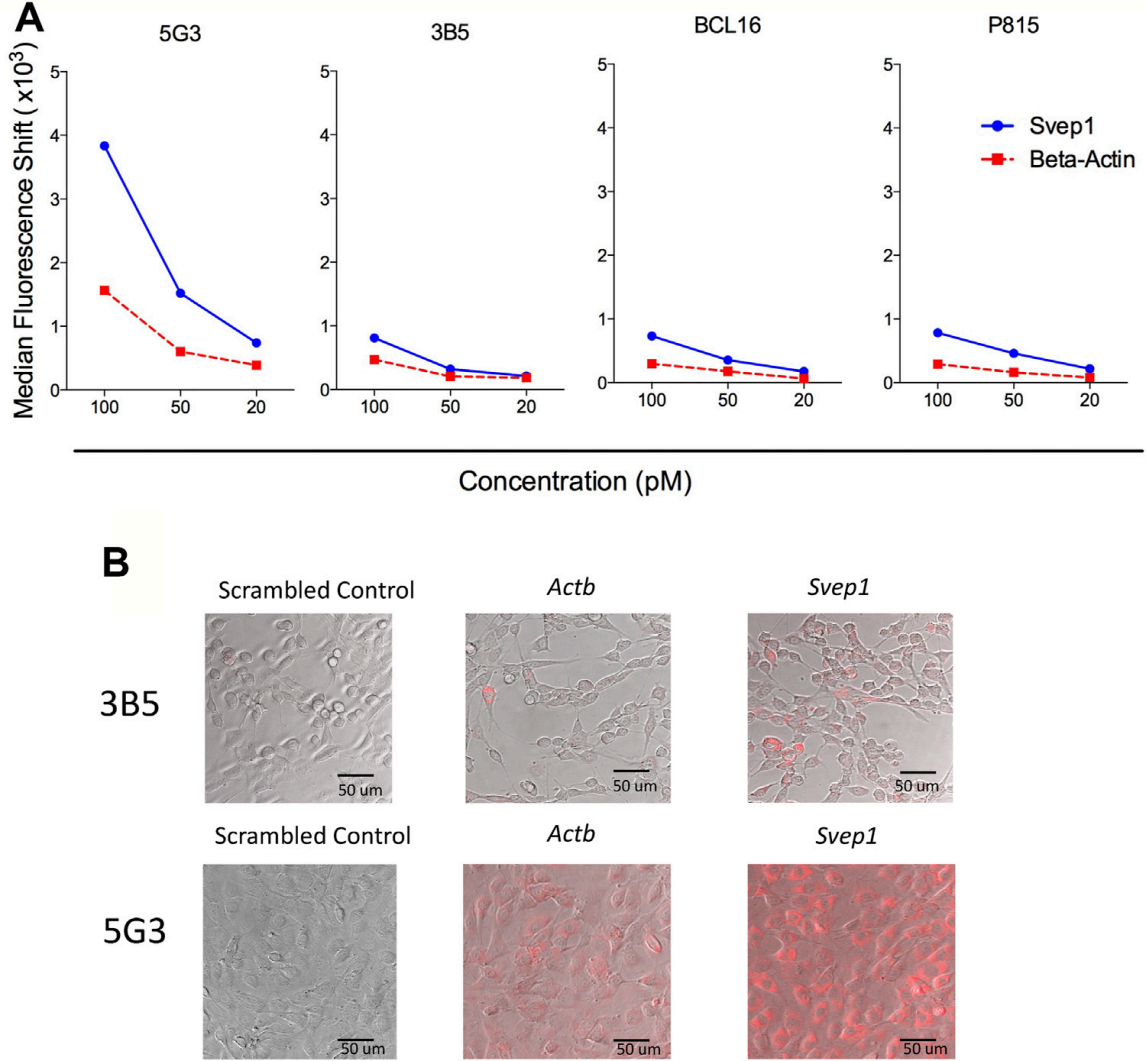
Homogeneous expression of *Svep1* by 5G3 stromal cells. **(A)** Cell lines were cultured to 80% confluency and then supplemented with SmartFlares ™ with the SmartFlare™ Scrambled Target Control Cy5, SmartFlare™ *Actb* Cy5 (housekeeping gene) or SmartFlare™ *Svep1* Cy5 (target gene) at concentrations of 100, 50 and 20 pM. After 16 h, flow cytometric analysis was performed, with the Scrambled Target Control used to set gates to identify fluorescence due to specific gene expression. Data are expressed as shift in median fluorescence above background. **(B)** Cells prepared in **(A)** were photographed by Confocal microscopy at 16 h after labelling.

Further attempts to identify *Svep1* expression on cells in the absence of specific antibodies, involved the use of SmartFlare™ probes combined with flow cytometry. Optimisation of the uptake method in terms of concentration and exposure time utilised several continuous cell lines; 5G3, 3B5, BCL1 and P815. Cells were cultured with the SmartFlare™ Scrambled Target Control and the specific *Svep1* or *Actb* control SmartFlare™ probe. This showed that only 5C3 and no other cell lines tested stained for *Svep1*, with low *Actb* staining. Optimal staining was obtained using 100pM of probe in a 16-hour uptake assay (Figure 5A). Confocal microscopy identified homogeneous fluorescent staining for *Svep1* in all 5G3 cells, with a lower level of *Actb* staining (Figure 5B). In contrast, 3B5 showed very few cells labelled for either marker. This method confirmed the uniform expression of *Svep1* across all 5G3 cells and with an absence of staining in 3B5 cells. *Svep1* is expressed by a range of tissue types, although any marker positive subsets within tissue are not known. Attempts to stain subsets isolated from dissociated tissues using SmartFlare™ probes gave limited success.

### Expression of *Svep1* by 5G3 Stroma is Related to Osteogenic Potential

**FIGURE 6:**
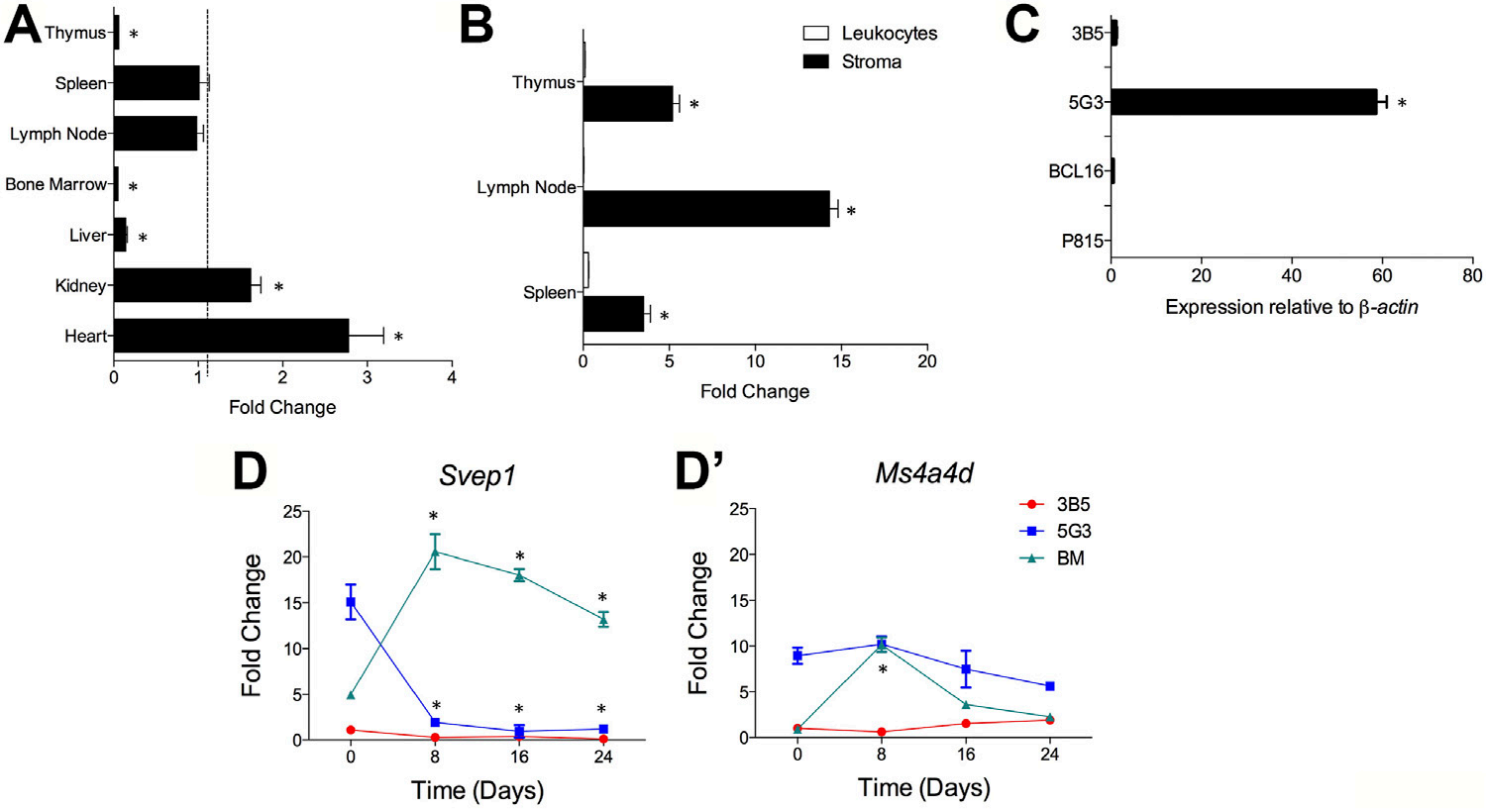
*Svep1* expression is restricted to stromal cells. The expression of *Svep1* was measured by qRT-PCR relative to *Actb* in each sample. Data are presented as mean ± S.E. for 4 independent PCR reactions. **(A)** *Svep1* expression was assessed in multiple organs and is presented in terms of fold change relative to spleen expression. Gene expression significantly different (*p* ≤ 0.05) to spleen is shown by *. **(B)** *Svep1* expression was compared in the stromal cell and leukocyte fractions prepared from lymphoid tissues. Significant difference (*p* ≤ 0.05) in gene expression between fractions is shown by *. Data represent *Svep1* expression in stromal cells relative to leukocytes for each organ. **(C)** Comparison is made of expression in several cell lines including 5G3 and 3B5 stromal lines, where 5G3 expression is significantly higher (*p* ≤ 0.05) than all others *. **(D)** Expression of *Svep1* is related to osteogenesis. The 5G3 and 3B5 cell lines were cultured for 28 days under mineralisation conditions which induce osteogenesis. *Svep1* expression **(D)** and the control gene *Ms4a4d* **(D’)** was measured at 8-day intervals. Controls included bone marrow progenitors induced to undergo osteogenesis over 24 days. Data represent the average fold change in gene expression in samples relative to 3B5 measured on Day 0. Gene expression significantly different (*p* ≤ 0.05) to the fold change value at Day 0 is shown by *.

*Svep1* expression was detected using qRT-PCR on cells from dissociated whole organs including thymus, spleen, lymph node, bone marrow, liver, kidney and heart. Expression was measured relative to *Actb* in triplicate reactions. Kidney and heart had the highest expression of *Svep1* at 1.62 and 2.78 fold greater than spleen (Figure 6A). In contrast, lymph node had a similar expression level to spleen, while liver, thymus and bone marrow showed 10-fold lower levels of *Svep1* expression (Figure 6A). Using qRT-PCR only stromal cells and not leukocytes in dissociated lymph node, thymus and spleen expressed *Svep1* (Figure 6B). Consistent with this is expression in 5G3 although not in 3B5 (Figure 6C). No expression was detected for the P815 mastocytoma line or the BCL1 B lymphoid cell line.

Previous studies have identified 5G3, as a stromal cell line with osteogenic differentiative potential (O’Neill et al., 2019). *Svep1* expression has been identified here as a marker of osteogenic progenitors which is lost on mineralisation. Following culture under mineralisation conditions, both 5G3 and 3B5 undergo osteogenesis with upregulation of genes like *Sp7* (Osterix), *Alp* (Alkaline phosphatase), *Oc* (Osteocalin), *Spp1* (Osteopontin) and *Bsp* (Bone sialoprotein). The expression of *Svep1,* and *Ms4a4d* (used as a control gene), was measured on 5G3 and 3B5 cells over 24-days of culture under mineralisation conditions. qRT-PCR was performed every 8 days to quantify gene expression. No *Svep1* or *Ms4a4d* gene expression was seen with the 3B5 control stromal line. Gene expression is therefore assessed relative to 3B5 cells at Day 0 (Figures 6D,D’). *Svep1* gene expression was readily detectable at Day 0 in 5G3 cells but reduced significantly to near zero by Day 8 as cells underwent mineralisation and became osteoblastic (Figure 6D). In contrast, *Ms4a4d* did not modulate expression in 5G3 under increasing mineralisation conditions (Figure 6D’). Both *Svep1* and *Ms4a4d* increased expression in BM-derived MSC by Day 8 when osteoprogenitors form, significantly reducing in level with mineralisation (Figures 6D,D’). *Svep1* and *Ms4a4d* therefore appear to be specific markers of stromal cells which are osteoprogenitors.

## Discussion

Stromal niches for HSC utilise multiple cell adhesion molecules. Since SVEP1 is an adhesion molecule that interacts with integrin α_9_β_1_ (Sato-Nishiuchi et al., 2012), a reduction in *Svep1* expression could prevent progenitors from binding to 5G3, and so reduce hematopoiesis and the development of myeloid cells within co-cultures. After *Svep1* shRNA was used to produce *Svep1* knockdown lines of 5G3 stroma, highly purified progenitor subsets were needed to show a change in the production of myeloid and DC subsets in co-cultures involving knockdown stroma. Selectins like SVEP1 would appear to anchor HSC on to stroma so ensuring their maintenance, quiescence, and supporting their later differentiation and mobilisation (Oh and Kwon, 2010).

The preparation of stable 5G3 knockdown cell lines was chosen over transient knockdown lines, since the co-culture system of HSPC over stroma assesses cell production over an extended period of 4 weeks. This strategy was applied for two reasons. Firstly, the stable transfectants produced are considered a heterogeneous population and the generation of a heterogeneous population is more time-efficient and achieves an average knockdown effect. However, this can lead to differences between experiments in the cell population produced over an extended culture period out to 28 days as seen in the two experiments shown here. Secondly, the generation of a single cloned knockdown cell line for each gene would be time-consuming and complex in terms of the assay methods needed to screen for single clonal transfectants. The knockdown procedure used here gave reduced expression of three genes, namely *Svep1*, *Csf1* and *Igf2*. The impact of knockdown was evident by differences in cell production and the clear capacity of shRNA transfected lines to reduce or change hematopoiesis.

Replicate co-cultures established with LT-HSC, MPP and MDP, showed reproducible changes in cell production within an experiment despite variation between experiments in terms of the type of cells produced. HSPC sorting procedures were held consistent between replicate experiments using equal numbers of mice and the same concentration of antibodies. Cell yield was always low, with variation in the number of LT-HSC and MPP recovered from each sort. Experiments were however standardised so that equal numbers of each progenitor type were added to each stromal type under test. Despite experimental difficulty, well controlled replicate experiments have been achievable.

Selectins and integrins are known to be essential in the hematopoietic process. For example, E-selectin expression by stroma drives HSC proliferation, such that a deficiency in E-selectin leads to HSC quiescence and greater capacity for HSC self-renewal (Winkler et al., 2012). P-selectin has been found to regulate myelopoiesis, such that transplantation of Lin^-^Sca1^+^cKit^+^ (LSK) cells from P-selectin^−/−^ mice produced higher numbers of myeloid progenitors *in vivo* compared with wild type controls (Sullivan et al., 2011). Based on the known function of selectins and integrins, it is hypothesised that SVEP1 adheres HSPC to 5G3 stroma *via* interaction with integrin α_9_β_1_. Once adherent, 5G3 may utilise signalling pathways that maintain HSPC and drive differentiation. If *Svep1* expression is reduced, HSPC may bind only weakly to stroma, leading to reduced differentiation and lower or delayed differentiation in co-cultures, perhaps with greater accumulation of progenitors.

The MDP population of Lin^-^Sca1^+^ckit^-^Flt3^+^CD115^+^ cells used here contains a progenitor/precursor of L-DC. It is not equivalent to the CX3CR1^+^ subset of these cells which was previously shown to lack progenitors of L-DC (Petvises and O’Neill, 2014a). Recent studies have also found that the MDP subset is not restricted in development to monocytes and DC as previously reported (Sathe et al., 2014). Agar colony assays performed on Lin^-^cKit^hi^Sca1^−^CD16/32^hi^CX_3_CR1^+^ MDP were unexpectedly found to give rise to granulocytes, and very few MDP produced both macrophages and DC in colony assays (Sathe et al., 2014). These studies by others raise doubt about the existence of a single progenitor within the MDP subset with restricted differentiative capacity for just monocytes and DC.

Since *Svep1* expression by 5G3 was directly linked to L-DC development, the possibility that *Svep1* encodes a specific marker for the *in vivo* cell equivalent to 5G3 was considered. In an attempt to identify *Svep1*-expressing cells *in vivo*, SmartFlare™ probes were employed. These served to show that all 5G3 cells expressed *Svep1.* Preliminary experiments using the SmartFlare™ technology indicated limitations for detecting *Svep1*-expressing cells *in vivo*. Several studies were therefore undertaken to assess the cell type in spleen which expresses *Svep1*. Expression was detected in several murine tissues by qRT-PCR, and shown to be restricted to the stromal fraction of lymphoid organs. This is consistent with earlier evidence that SVEP1 is a cell surface protein on mesenchymal and osteoblastic cells (Shur et al., 2006; Sato-Nishiuchi et al., 2012). It was also found to be specific to resting 5G3 cells, and was lost upon culture under mineralisation conditions which induce osteogenesis. *Svep1* expression therefore appears to be limited to stromal cells with osteoprogenitor characteristics, consistent with the phenotype of 5G3 as a perisinusoidal/perivascular reticular cell (Periasamy et al., 2018).

These results identify SVEP1 as another potential adhesion pathway for maintenance of self-renewing HSPC to hold them in close proximity with stromal cells (Martinez-Agosto et al., 2007; Chen et al., 2013). It is also well known that integrin-ligand binding generates intracellular signals that result in changes in gene expression, cell proliferation, survival and differentiation (Legate et al., 2009). SVEP1 is therefore potentially a very important regulator of stem/progenitor cell self-renewal and may have future therapeutic importance in terms of manipulation or supplementation of hematopoiesis.

Although IGF2 is a growth factor for HSC, knockdown of *Igf2* did not significantly impact L-DC production and gave only a partial or transitory knockdown effect on cDC-like cell production. Previously it was shown to weakly inhibit production of cDC-like cells developing from myeloid progenitors (Petvises and O’Neill, 2014a). IGF2 has been shown to be important in the maintenance and expansion of early HSC in the aorta gonad mesonephros (AGM), the fetal liver and in bone marrow (Zhang and Lodish, 2004; Mascarenhas et al., 2009). However, IGF2 function is not restricted to one cell type, and the IGF1R receptor which binds IGF2 is commonly expressed by numerous cell types (Brown et al., 2008; Chao and D’Amore, 2008). Interaction of IGF2 with IGF1R leads to phosphorylation of signalling molecules including mitogen-activated protein kinase and protein-kinase B, which function together to drive cell survival and differentiation (Prince et al., 2007). In co-cultures shown here involving LT-HSC and MDP, knockdown of *Igf2* in 5G3 stroma gave a reduction in production of only cDC-like cells.

CSF1 was also investigated as a regulator of *in vitro* hematopoiesis and is strongly expressed by both 5G3 and 3B5. CSF1 was originally described as a promoter of monocyte and DC development (MacDonald et al., 2005; Hume and MacDonald, 2012). However, here it is more specifically shown to act as a factor driving a subset of progenitors within the MPP population, such that when *Csf1* was knocked down in 5G3, a subset of MPP proceeded towards L-DC development. Evidence for a decrease in the development of cDC-like cells due to CSF1 inhibition serves to reinforce former evidence that CSF1 is a growth factor required for production of cDC-like cells rather than L-DC in *in vitro* co-cultures (Petvises and O'Neill, 2014; Petvises and O’Neill, 2014a). One explanation for this result is that CSF1 may be important for self-renewal or proliferation of a subset of MPP progenitors, although not the L-DC progenitors, such that knockdown of CSF1 gave preferential production of L-DC. The importance of CSF1 in cDC development *in vivo* has also been reported previously such that the administration of CSF1 to *Flt3* ^*−/−*^ mice increases cDC numbers by 2-fold in spleen, confirming that cDC development depends on CSF1 (Fancke et al., 2008).

Delineation of molecules here represents a significant contribution to the characterisation of hematopoietic niches, how they function and how they are regulated. The identification of stromal cells, growth factors and molecules that contribute to the hematopoietic process, brings us closer to the realm of regulating hematopoiesis *in vivo*, and to inhibiting niches which support cancer stem cells. In terms of the therapeutic importance of the findings made here, it is important to emphasise that the knockdown studies described here directly link gene expression to the function of SVEP1 in early hematopoiesis involving HSPC. The finding that a subset of human bone marrow stroma expresses *Svep1* (Kuçi et al., 2010; Kuçi et al., 2019), emphasises the significance of this result. These cells represent the perivascular reticular cells of the HSC niche in human bone marrow. In future it will be very important to investigate more fully the expression and function of SVEP1 by human bone marrow and spleen stromal subsets, and to determine whether changes in SVEP1 expression is associated with myeloid leukemias or myeloproliferative disorders. The opportunity exists to identify molecules as potential regulators of myelopoiesis. Molecular mimics of SVEP1 could be used therapeutically to enhance myelopoiesis, and inhibitors of SVEP1 binding to HSPC could be used to treat myeloproliferative disorders and leukemia.

## Conclusion

This study identifies SVEP1 expressed by stromal cells in spleen as an important regulator of hematopoiesis in spleen. In well-controlled experiments, *Svep1*, *Csf1* and *Igf2* knockdown cell lines of the 5G3 mesenchymal stromal line were co-cultured with purified subsets of bone marrow-derived HSPC. These experiments identified *Svep1* as critical to the development of L-DC from progenitors from sorted populations of LT-HSC, MPP and also the MDP from bone marrow. *Csf1* and *Igf2* knockdown was effective in reducing the low level production of cDC-like cells from progenitors within the MDP subset. *Csf1* knockdown was found to enhance the production of L-DC probably through indirect inhibition of a subset of myeloid progenitors within the MPP subset.

## Data Availability

The raw data supporting the conclusion of this article will be made available by the authors, without undue reservation.
